# The relationship of atrial fibrillation with left atrial size in patients with essential hypertension

**DOI:** 10.1038/s41598-024-51875-1

**Published:** 2024-01-13

**Authors:** Rami Saadeh, Bara Abu Jaber, Taqwa Alzuqaili, Sara Ghura, Taiba Al-ajlouny, Abdallah M. Saadeh

**Affiliations:** 1 Department of Public Health and Community Medicine, Faculty of Medicine, Jordan University of Science and Technology, Irbid, 22110 Jordan; 2 Department of Internal Medicine, King Abdullah University Hospital, Irbid, 22110 Jordan; 3 Faculty of Medicine, Jordan University of Science and Technology, Irbid, 22110 Jordan; 4 Department of Internal Medicine, Faculty of Medicine, Jordan University of Science and Technology, Irbid, 22110 Jordan

**Keywords:** Cardiology, Cardiovascular diseases

## Abstract

Atrial fibrillation (AF) is the most common sustained arrhythmia and it is a major public health problem worldwide. Hypertension is one of the major risk factors for the development of AF. This study is carried out to determine the prevalence and independent risk factors for atrial fibrillation (AF) in hypertensive patients and to evaluate the relationship of AF with left atrial size. This is a retrospective observational cross – sectional study that used a retrospective electronic chart review of all admitted patients to cardiology department at King Abdullah university hospital (KAUH) in Irbid, Jordan, with a diagnosis of hypertension along with various acute cardiac admissions, including AF during 1-year period (January 1st to December 31 of 2021). Risk factors for AF (age, sex, DM, coronary artery disease, valvular heart disease, Cor-pulmonale, obstructive sleep apnea, and congestive cardiac failure) were retrieved from electronic charts of the patients. A total of 958 patients were admitted to the coronary care unit (CCU) and intermediate care unit (IMCU) during a 1-year period. Among them, 276 had 2 or 3 admissions. The main reason of admission was acute coronary syndrome (n = 491), heart failure (n = 180), and AF (n = 144), indicating AF prevalence of 15%. However, there were 40 patients with combined causes. All patients in the study (n = 958) were diagnosed with hypertension, including patients with atrial fibrillation (n = 144). The mean age of patients was 61.4 (± 11.46) years, and approximately two thirds of them were males (65.4%). The binary logistic regression model demonstrated a significant statistical relationship of age, left atrial size, coronary artery disease, left ventricular ejection fraction, left ventricular dimensions in systole and diastole, and heart failure with the occurrence of AF after controlling for gender, smoking, and diabetes. Findings indicate that left atrial size plays a significant role in the development of AF in patients with hypertension. However, the prevalence of AF significantly increased with advancing age in both sexes because of increased left ventricular hypertrophy, which leads to increased left atrial size.

## Introduction

Atrial fibrillation (AF) is the most common sustained arrhythmia and it is a major public health problem worldwide associated with substantial morbidity and mortality^1,2^. Risk factors for atrial fibrillation include age, hypertension, coronary artery disease, diabetes mellitus (DM), chronic obstructive pulmonary disease (COPD), obstructive sleep apnea (OSA), valvular heart disease, and congestive heart failure^3–6^.

Among these risk factors, hypertension is the most common cause of AF because hypertension is the most prevalent disease among these conditions^2,7–9^. In some studies, up to 90% of AF patients are observed to be hypertensive^10^. However, the mechanisms underlying the increased susceptibility to atrial fibrillation in hypertensive patients are not completely understood. Nonetheless, studies that illustrated the development of AF in various risk factors indicated the occurrence of pathophysiological changes in the atrium at a cellular and molecular level, leading to interstitial fibrosis in the atrial tissue. The remodeling of the atrial structure increases the propensity to re-entry atrial tachyarrhythmia^11–15^.

The renin–angiotensin–aldosterone system (RAAS) may play a role in the occurrence of AF. Angiotensin-II has been shown to regulate cardiac cell proliferation and modulating myocyte ion channels^8^. Thus, the use of angiotensin converting enzyme (ACE) inhibitors and Angiotensin-II receptor blockers (ARBs) may be effective in the prevention of AF in patients with heart failure^8,16,17^. However, it is difficult to determine if these agents are effective in the prevention of AF in patients with hypertension. This study aims to investigate the relationship of LA size with the occurrence of AF in hypertensive patients.

## Methods

### Study design

This was a retrospective observational cross – sectional study that reviewed records of patients diagnosed with hypertension and admitted to the cardiology department of King Abdullah university hospital (KAUH) in Irbid, Jordan from 1/1/2021 to 31/12/2021. The diagnosis of AF and associated risk factors were obtained from patient’s electronic records. Records included the Electrocardiographic reports (ECG’s), laboratory data, echocardiographic reports and clinical data (history and progress notes). Left atrium (LA) size was measured by transthoracic echocardiography machine (Hp-Sonos 5500, USA), measuring anteroposterior dimensions by M-mode directed by two dimensional (2-D) (real-time) echo in long axis parasternal view measured from LA posterior wall leading edge to leading edge at the level of aortic sinuses.

### Study participants

All patients with hypertension who were admitted to the cardiac units (CCU and IMCU) of KAUH during January to December of 2021 were included. Diagnosis of hypertension was documented from electronic charts based on history of hypertension and being on antihypertensive treatment. Presence of AF was documented from the electrocardiograms and retrieved from patients’ electronic charts and the progress notes of the patients during hospitalization or during the follow up in the outpatient clinics.

### Inclusion and exclusion criteria

Only AF in setting of hypertension was included in this study (144 cases). AF due to rheumatic valvular heart disease (3 cases), pre-excitation (2 cases), AF associated with thyrotoxicosis (1 case), AF associated with chronic Cor pulmonale (2 cases) and AF associated with obstructive sleep apnea (OSA; 1 case) were excluded from the study population, because these cases were not having hypertension.

### Study variables

Patients’ gender, age, and smoking status were reported. Further, patients’ records of average systolic blood pressure, diastolic blood pressure, left atrial size, left ventricular ejection fraction (%), left ventricular dimensions in diastole, left ventricular dimensions in systole, and serum creatinine (µmol/L) were included.

Risk factors for AF (age, sex, diabetes mellitus, coronary artery disease, dilated cardiomyopathies (DCM), congenital heart disease, valvular heart disease, Cor-pulmonale and congestive cardiac failure) were also retrieved from electronic charts of the patients and registered on an excel sheet.

### Analysis

The baseline characteristics of the patients with atrial fibrillation and those without were compared using students *t* test for continuous variables, and chi-square test (X^2^) for categorical variables. Furthermore, binary logistic regression was used to predict the occurrence of atrial fibrillation based on age, left atrial size, coronary artery disease, left ventricular ejection fraction, left ventricular dimensions in systole and diastole, and heart failure with the occurrence of atrial fibrillation after controlling for gender, smoking, and diabetes. Analysis was performed using the statistical package (“SPSS” software version 23), and a p-value less than 0.05 was considered the level of significance.

### Ethics approval and consent to participate

This study was approved by the institutional review board of Jordan University of Science and Technology (number 124-2022). The requirement for informed consent from the study subjects was waived by the IRB of (Jordan University of Science and Technology/Research committee) due to the retrospective study design. Only patients’ file number were extracted with the data and no names or identifiable information was included. In addition, the committee ensured that all methods used in this research was performed in accordance with relevant guidelines/regulations.

## Results

There was a total of 958 hypertensive patients included in the study. The mean age of study patients was 61.40 (± 11.46) years, most of them were males (65.4%), and 40.3% were current smokers. There was a high prevalence of coronary artery disease (CAD) and diabetes mellitus (DM) among participating patients (51.3% and 59.2%, respectively) and about 1 out of each 5 patients had HF (Table 1).

**Table 1:** Demographic and other characteristics of patients.

Characteristics	Atrial fibrillation	No atrial fibrillation	Total*	P-value
Female	68 (20.5%)	263 (79.5%)	331 (34.6%)	**0.001**
Male	76 (12.1%)	551 (87.9%)	627 (65.4%)	
Total	144	814	958	
Age	(67.97 ± 10.68)	(60.24 ± 11.21)	(61.40 ± 11.46)	** < 0.001**
Smoking (yes)	56 (14.5%)	330 (85.5%)	386 (40.3%)	0.71
Smoking (no)	88 (15.4%)	484 (84.6%)	572 (59.7%)	
Total	144	814	958	
Coronary artery disease (yes)	97 (19.8%)	394 (80.2%)	491 (51.3%)	** < 0.001**
Coronary artery disease (no)	47 (10.1%)	420 (89.9%)	467 (48.7%)	
Total	144	814	958	
Heart failure (yes)	74 (41.1%)	106 (58.9%)	180 (18.8%)	** < 0.001**
Heart failure (no)	70 (9.0%)	708 (91.0%)	778 (81.2%)	
Total	144	814	958	
Diabetes (yes)	89 (15.7%)	478 (84.3%)	567 (59.2%)	0.488
Diabetes (no)	55 (14.1%)	336 (85.9%)	391 (40.8%)	
Total	144	814	958	
Systolic blood pressure	(131.27 ± 20.55)	(133.13 ± 19.04)	(132.85 ± 19.28)	0.313
Diastolic blood pressure	(76.33 ± 10.13)	(78.09 ± 10.61)	(77.82 ± 10.55)	0.066
Left atrial size	(4.2 ± 0.461)	(3.91 ± 0.297)	(3.96 ± 0.342)	** < 0.001**
Left ventricular ejection fraction (%)	(47.85 ± 11.63)	(52.0 ± 9.0)	(51.40 ± 9.55)	** < 0.001**
LV dimension in diastole	(5.32 ± 0.537)	(5.17 ± 0.491)	(5.19 ± 0.501)	**0.002**
LV dimension in systole	(4.04 ± 0.732)	(3.85 ± 0.612)	(3.88 ± 0.634)	**0.005**
Serum Creatinine µmol/L	(141.21 ± 118.0)	(118.32 ± 126.45)	(121.41 ± 125.04)	**0.044**

Categorical variables were analyzed using chi – square test of independence.

Continuous variables were analyzed using student t-test.

*The percentage in total column represents total percentage of each row.

Significant values are in bold.

Patients with AF represented 15% of the sample (n = 144). Among AF patients, there were 6 patients (4.2%) with valvular health diseases, 10 (6.9%) with Cor pulmonale or COPD, and 6 patients (4.2%) with obstructive sleep apnea (OSA). Those patients had only one of these condition, except 3 patients who had both Cor pulmonale and OSA.

There was a significant statistical difference between patients with AF and patients without AF for the means of the following measured variables: LA size, LVEF%, LV dimensions in diastole and systole (P-values < 0.05). Serum creatinine mean levels were not statistically different between patients with AF and those without AF (Table 1).

The binary logistic regression model demonstrated a significant relationship of age, LA size, CAD, and HF with the occurrence of AF after controlling for gender, smoking, and diabetes. For each year increase in age, the probability of AF occurrence increased by 4.7% [OR = 1.047, 95% CI   1.026–1.069, p = value < 0.0001]. In addition, the risk of AF increased 3.2 times more among patients with increased LA size [OR = 3.204, 95% CI   1.749–5.870, p = value < 0.0001]. There was an increased risk of AF by 16% for each 1 cm increase in LA size, as noticed in Table 2.

**Table 2:** The relationship of atrial fibrillation with variables of interest using binary logistic regression.

Parameter	Estimate	Standard error	Odds ratio	Wald 95% confidence limits		P value
LVEF% (average)	– 0.033	0.024	0.968	0.923	1.015	0.183
LV dimension in diastole	– 0.244	0.427	0.784	0.340	1.808	0.568
LV dimension in systole	– 0.741	0.417	0.476	0.210	1.079	0.076
Age	0.046	0.011	1.047	1.026	1.069	< 0.0001
LA size (largest)	1.164	0.309	3.204	1.749	5.870	< 0.0001
Coronary artery disease	0.722	0.227	2.058	1.319	3.211	0.001
Heart failure	1.743	0.267	5.715	3.389	9.637	< 0.0001

The model controlled for the following variables: gender, smoking, and diabetes.

The reasons of admission to cardiac units (CCU and IMCU) during the year (January 1st to December 31st of 2021) are illustrated in Table 3.

**Table 3:** Reasons of patients’ admissions in the cardiac units of KAUH.

Reason of admission – all causes (n = 958)	Acute coronary syndrome (ACS)	N = 491
	Heart failure (HF)	N = 180
	Atrial fibrillation	N = 144
	Supraventricular tachycardia (VT)	N = 75
	Hypertensive crises or uncontrolled hypertension	N = 22
	Ventricular premature contraction	N = 16
	Complete heart block or mobitz type 2 heart block	N = 12
	HF + AF	N = 9
	Uncontrolled BP with HF	N = 5
	VT with HF	N = 4
Total hypertensive patients		N = 958
Re-admissions: twice or 3 times		N = 276

## Discussion

Atrial fibrillation is associated with several risk factors and predisposing conditions that increases its occurrence, including hypertension that accelerates the development and progression of AF^18^. The prevalence of AF in this current study was 15%. This rate is similar to a study that showed prevalence of AF in hypertensive patients was equal to 14.9%. In that study, there were 9474 hypertensive patients followed for a median period of 24.1 years and a total of 1414 cases of AF (14.9%) was identified during the follow-up period^9^.

Most of the studies has identified hypertension as a major contributor to the development of AF, and therefore, the study population of this study were all hypertensive patients in order to identify other intrinsic cardiac conditions that could affect the occurrence of AF. The American Heart Association (AHA) listed factors that are associated with AF development, including advanced age, a prolonged uncontrolled hypertension, underlying heart disease such as valve problems or hypertrophic cardiomyopathy, family history, and other chronic conditions like diabetes or sleep apnea^19^. In the current study, age, heart disease, CAD, and LA size were significantly associated with a higher likelihood of AF occurrence among hypertensive patients. Similarly, the Framingham heart study reported that the incidence of AF increased with age, heart failure, coronary heart disease, among other factors. AF was almost doubled every 10-year increment in age. The significant relationship of age with AF was shown in other studies as well^20,21^. In addition, the relationship of coronary heart disease and heart failure with AF was well established in previous Framingham heart studies and other studies, which was significant even with adjustment for age and gender^18^.

There are few studies that investigated the relationship of atrial or ventricular size with the occurrence or the progression of AF. However, Kannel et al. in 2 scoping review studies illustrated that echocardiographic predictors of AF included left atrial enlargement, left ventricular fractional shortening, left ventricular wall thickness, and mitral annular calcification^5,18^. Left atrial size was an independent predictor for persistent AF, even when factors like age and gender were controlled^7,22^, which is similar to the findings of the current study. Further, Gerdts et al. had demonstrated that left ventricular hypertrophy and LA enlargement attributed to higher rate of AF in hypertensive patients^23^.

The occurrence of AF seems to be complex in nature due to the several risk factors that could play a role and impose a multifactorial effect on the disease. Moreover, the structural intrinsic changes of the heart that is likely to progress with the advancement of age contribute to the development of AF. While a patient with chronic conditions, such as diabetes or hypertension, advances in age, progressive changes manifest in LA anatomy and function, may promote AF^8^.

Various inflammatory markers and mediators such as C-reactive protein (CRP), tumor necrosis factor (TNF-a), interleukin -2 (IL-2), interleukin – 6 (IL-6), interleukin – 8 (IL-8), and monocyte chemoattractant protein—I (MCP – I) have been linked to the development and outcome of AF^15,24–28^.

Animal models and human studies revealed a strong relationship between atrial myopathy and incidence of AF. Atrial myopathy as characterized by atrial fibrotic remodeling leading to electrical and autonomic changes which facilitate the development of AF^29^. Atrial myopathy leads to structural and electrophysiological changes of the left atrium (i.e. inflammation, oxidative stress, stretch, and fibrosis) which in turn progress into electrical and autonomic remodeling and prothrombotic state^29^.

Diagnosis of atrial myopathy entails the usage of several tools, such as electrical (ECG), echocardiography, laboratory tests (inflammatory biomarkers), tissue biopsy, and 4 – dimensional magnetic resonance imaging (4-D MRI). The clinical implications of diagnosing atrial myopathy by these means may assist clinicians to consider anticoagulation therapy, even before the onset of AF, for selected subgroups of patients with low risk of strokes. Therefore, Determining the LA size, echocardiographically, can be considered a clinical risk identifier in preclinical AF which should be included in routine comprehensive echocardiography evaluation^27^.

### Study limitations

Since this study is a retrospective study, we could not identify paroxysmal AF from chronic permanent AF. Another limitation is that we were not able to include the left ventricular mass index because the diastolic posterior wall thickness was not measured in the majority of patients.

## Conclusion

Findings suggest that LA enlargement in hypertensive patients play a major role in the development of AF through structural and electrophysiological changes to the atrium and other possible mechanisms^21^. Although there are several risk factors associated with the development of AF, chronic conditions and advancement of age remains the most critical detrimental factors in the onset and progression of AF. Moreover, large scale randomized studies are needed to establish a definitive role of antihypertensive therapy in reducing AF incidence.

## Data Availability

The datasets used and/or analyzed during the current study available from the corresponding author on reasonable request.

## References

[1] Benjamin EJ (1994). Independent risk factors for atrial fibrillation in a population-based cohort. The Framingham Heart Study. JAMA.

[2] Ogunsua AA, Shaikh AY, Ahmed M, McManus DD (2015). Atrial fibrillation and hypertension: Mechanistic, epidemiologic, and treatment parallels. Methodist Debakey Cardiovasc. J..

[3] Healey JS, Connolly SJ (2003). Atrial fibrillation: Hypertension as a causative agent, risk factor for complications, and potential therapeutic target. Am. J. Cardiol..

[4] Huxley RR (2011). Absolute and attributable risks of atrial fibrillation in relation to optimal and borderline risk factors: The Atherosclerosis Risk in Communities (ARIC) study. Circulation.

[5] Kannel WB, Wolf PA, Benjamin EJ, Levy D (1998). Prevalence, incidence, prognosis, and predisposing conditions for atrial fibrillation: Population-based estimates 1. Am. J. Cardiol..

[6] Psaty BM (1997). Incidence of and risk factors for atrial fibrillation in older adults. Circulation.

[7] Aronow WS (2017). Hypertension associated with atrial fibrillation. Ann. Transl. Med..

[8] Verdecchia P, Angeli F, Reboldi G (2018). Hypertension and atrial fibrillation. Circ. Res..

[9] Liao LZ, Wen XY, Zhang SZ, Li WD, Zhuang XD (2021). Hypertension and atrial fibrillation: A study on epidemiology and mendelian randomization causality. Front. Cardiovasc. Med..

[10] Widimský J (2012). Arterial hypertension and atrial fibrillation: Selecting antihypertensive therapy. Cor et Vasa.

[11] Okin PM (2010). Relationship of left atrial enlargement to persistence or development of ECG left ventricular hypertrophy in hypertensive patients: Implications for the development of new atrial fibrillation. J. Hypertens..

[12] Samanta R, Pouliopoulos J, Thiagalingam A, Kovoor P (2016). Role of adipose tissue in the pathogenesis of cardiac arrhythmias. Heart Rhythm.

[13] Schotten U, Verheule S, Kirchhof P, Goette A (2011). Pathophysiological mechanisms of atrial fibrillation: A translational appraisal. Physiol. Rev..

[14] Toh N (2010). Left atrial volume combined with atrial pump function identifies hypertensive patients with a history of paroxysmal atrial fibrillation. Hypertension.

[15] Nattel S (2017). Molecular and cellular mechanisms of atrial fibrosis in atrial fibrillation. JACC Clin. Electrophysiol..

[16] Iravanian S, Dudley SC (2008). The renin-angiotensin-aldosterone system (RAAS) and cardiac arrhythmias. Heart Rhythm.

[17] Mascolo A (2021). The role of renin-angiotensin-aldosterone system in the heart and lung: Focus on COVID-19. Front. Pharmacol..

[18] Kannel WB, Benjamin EJ (2008). Status of the epidemiology of atrial fibrillation. Med. Clin. N. Am..

[19] American Heart Association (AMA). *Who is at Risk for Atrial Fibrillation?* https://www.heart.org/en/health-topics/atrial-fibrillation/who-is-at-risk-for-atrial-fibrillation-af-or-afib (2023).

[20] Heeringa J (2006). Prevalence, incidence and lifetime risk of atrial fibrillation: The Rotterdam study. Eur. Heart J..

[21] Wasmer K, Eckardt L, Breithardt G (2017). Predisposing factors for atrial fibrillation in the elderly. J. Geriatr. Cardiol. JGC.

[22] van de Vegte YJ, Siland JE, Rienstra M, van der Harst P (2021). Atrial fibrillation and left atrial size and function: A Mendelian randomization study. Sci. Rep..

[23] Gerdts E (2002). Correlates of left atrial size in hypertensive patients with left ventricular hypertrophy: The Losartan Intervention For endpoint reduction in hypertension (LIFE) study. Hypertension.

[24] Chung MK (2001). C-reactive protein elevation in patients with atrial arrhythmias: Inflammatory mechanisms and persistence of atrial fibrillation. Circulation.

[25] Deng H (2011). Role of tumor necrosis factor-alpha in the pathogenesis of atrial fibrillation. Chin. Med. J..

[26] Friedrichs K, Klinke A, Baldus S (2011). Inflammatory pathways underlying atrial fibrillation. Trends Mol. Med..

[27] Guo Y, Lip GY, Apostolakis S (2012). Inflammation in atrial fibrillation. J. Am. Coll. Cardiol..

[28] Patel P, Dokainish H, Tsai P, Lakkis N (2010). Update on the association of inflammation and atrial fibrillation. J. Cardiovasc. Electrophysiol..

[29] Shen MJ, Arora R, Jalife J (2019). Atrial myopathy. JACC Basic Transl. Sci..

